# MASTer cell: chief immune modulator and inductor of antimicrobial immune response

**DOI:** 10.3389/fimmu.2024.1360296

**Published:** 2024-04-04

**Authors:** Tomás Alejandro Suárez Vázquez, Nallely López López, Mario César Salinas Carmona

**Affiliations:** Department of Immunology, School of Medicine and Dr. Jose Eleuterio Gonzalez University Hospital, Universidad Autónoma de Nuevo León, Monterrey, Mexico

**Keywords:** mast cell, immunomodulation, bacterial infections, innate immunity, degranulation

## Abstract

Mast cells have long been recognized for their involvement in allergic pathology through the immunoglobulin E (IgE)-mediated degranulation mechanism. However, there is growing evidence of other “non-canonical” degranulation mechanisms activated by certain pathogen recognition receptors. Mast cells release several mediators, including histamine, cytokines, chemokines, prostaglandins, and leukotrienes, to initiate and enhance inflammation. The chemical nature of activating stimuli influences receptors, triggering mechanisms for the secretion of formed and new synthesized mediators. Mast cells have more than 30 known surface receptors that activate different pathways for direct and indirect activation by microbes. Different bacterial strains stimulate mast cells through various ligands, initiating the innate immune response, which aids in clearing the bacterial burden. Mast cell interactions with adaptative immune cells also play a crucial role in infections. Recent publications revealed another “non-canonical” degranulation mechanism present in tryptase and chymase mast cells in humans and connective tissue mast cells in mice, occurring through the activation of the Mas-related G protein–coupled receptor (MRGPRX2/b2). This receptor represents a new therapeutic target alongside antibiotic therapy. There is an urgent need to reconsider and redefine the biological role of these MASTer cells of innate immunity, extending beyond their involvement in allergic pathology.

## Introduction

Mast cells (MCs) are critical in the innate immune response beyond their well-known allergy involvement. In recent years, research on MCs has led to several important consensus findings regarding their direct or indirect participation in the host defense against bacteria and their role in immune responses (1–7). MCs acting as sentinels in immunosurveillance are responsible for monitoring interfaces directly exposed to the external environment, such as skin and mucous membranes. When they detect bacterial invaders through their pathogen recognition receptors (PRRs), these cells initiate an immune response that releases a wide range of mediators depending on the offending microbes (8–11). This innate immune response contains the infection by direct mechanisms, such as phagocytosis, reactive oxygen species (ROS) production, extracellular trap formation, and secretion of antimicrobial peptides (AMPs) known as classical or canonical mechanisms (12–15), or non-cannocical mechanisms, such as recruitment, activation, and modulation of immune cells (16, 17).

In this work, we address how MCs influence the immune response by interacting with other cells, modulating their activity on the role of MCs in the fight against bacterial infections. New therapeutic approaches targeting MCs and controversies regarding the potential benefits of modulating MC physiology are also discussed.

## Mast cell biology

MCs are key elements of the innate immune system. They were first described in 1878 by Paul Ehrlich, who defined their chemical and histological properties using basic aniline dyes. He described metachromasia with these dyes and found them in connective tissue zones (18). Later, it was determined that the metachromatic nature of the granules present in these cells was due to the interaction of aniline with heparin in the granules (19). In addition to his discovery, Ehrlich coined the famous term “Mastzellen” to name these metachromatic cells, where the German word “mast” denotes a fattening function, alluding to their peculiar intracellular structures in the form of granules. He hypothesized about their nutritional function (20).

MCs were later found to be of hematopoietic lineage because, like any other population of the immune system, these cells have a CD45+ phenotypic marker indicating their bone marrow origin. However, these cells were found in the intestine, lung, peritoneum, skin, etc., and were considered tissue-resident cells for a long time. In 1977, Kitamura’s experiments demonstrated their bone marrow origin (21). Ribatti and Crivellato found that transplanting bone marrow cells from wild-type (WT) mice to MC-deficient W/Wv mice proved that MCs originate from precursors that reside in the bone marrow (20). Ontogeny studies of MCs indicate several distinct spatiotemporal stages. The first hematopoietic and erythromyeloid progenitors (EMPs) emerge in the extra-embryonic yolk sac (YS). The second stage of hematopoietic stem cells (HSCs) occurs in the aorta-gonad-mesonephros; at this stage, MC precursors present a CD34+/CD117+ phenotype, the same as basophil progenitors (22). EMPs in adult mice are maintained in the adipose tissue and function as a source of stem cell precursors for MC progenitors in the skin (23), a remarkable difference with mucosal MCs (MMCs), which originate from fetal HSCs, continuing their replacement from bone marrow.

The replacement of YS-derived connective tissue-type MCs (CTMCs) occurs with tissue-specific kinetics, maintaining their independent origin in adult HSCs throughout life (24). They are distinct from definitive adult MCs regarding their phenotype and gene expression profile (25, 26). This information clarifies two independent origins of MCs in the adult: a subpopulation of MCs (CTMCs) derived from the (YS) remains in the skin and is renewed from local clonal precursors. The second subpopulation is located in the mucosa (MMC) and is renewed from bone marrow. Both MC subpopulations have been described intensively in allergic diseases, and their role in bacterial infection is little known. We now know that, despite having a hematopoietic origin, MCs become tissue-resident cells at later stages of maturation, where, upon entry into the tissue, local expansion of these progenitors results in the formation of clones covering territories that are remarkably stable over time, possibly for the entire life of the animal, providing local maintenance of the MC population without the need for new bone marrow precursors (26).

The c-kit cellular receptor of MCs explains the mechanism that maintains this cell population out of circulation. Its ligand, the stem cell factor, provides the necessary stimuli to induce differentiation and expression of surviving signaling. The c-kit cellular receptor, also known as CD117, is present only in mature MCs and is expressed on HSCs and retained throughout their development and differentiation; however, it is downregulated during the differentiation of other bone marrow–derived cells, including basophils that reach their maturation in the bone marrow before being released into the blood.

The basophil and MC progenitor is identified by CD34+ and the CD117+ surface markers. During the differentiation of this progenitor, the CD34 marker disappears and retains only CD117. In addition to this state of differentiation, FcϵRI+ expression appears on MCs. On the other hand, basophils stop expressing CD34+ and CD117+ and acquire FcϵRI+ during their differentiation process. Both MCs and basophils share the FcϵRI+ that will remain during their lifetime. This divergence, phenotypic recognition, and specialized functions of both lineages determine the roles of basophils and MCs in homeostasis and their involvement in some pathological conditions (20). The very few circulating MC progenitors (MCPs) in the blood eventually enter the peripheral tissues and serosal cavities, where they complete their maturation. The presence of proteoglycans such as chondroitin sulfate (MMCs) and heparin (CTMCs) characterize the MC phenotype in mice (27). In addition, protease content such as tryptase (MCT), chymase (MCC), and coexistence tryptase and chymase (MCTC) are also helpful in identifying the tissue origin of MCs in humans (28–30).

## Canonic and non-canonical mast cell activation

Traditionally, the key players in the allergy mechanism that triggers inflammation appear during the interaction between IgE and the IgE high-affinity receptor (FcϵRI). Specific antigens recognized by the antigen-binding (Fab) region of the IgE antibody present in the MC surface initiate the subsequent cascade of signaling events downstream of the receptor. The antigen (allergen)–IgE interaction triggers MC degranulation, leading to an allergy response (31). This degranulation releases a significant amount of biologically active inflammatory mediators, leading to harmful effects that can trigger anaphylaxis, all occurring within seconds. MCs have been considered for many years as a cellular paradox because they are not evolutionarily useful for the organism to go into shock from innocuous substances; however, new evidence shows the beneficial role of MCs and their contribution to host defense against pathogens (32). It is likely more relevant in infection control than just playing a detrimental role in allergy. Other studies, including mice models of *Trichinella spiralis* infection, demonstrated the relevant role of IgE and MCs in clearing this infection (33).

Recent publications have also provided evidence of the protective mechanisms against other pathogens. In a mouse *Listeria monocytogenes* infection model, it was observed that TLR2, responsible for the activation of MCs, plays a crucial role in regulating the synthesis of IL-6 and IL-13 during the innate immune response to this bacterium (34).

Using a *S. aureus* skin infection model, Starkl et al. found that IgE antibody production contributes to bacterial clearance, improving the systemic host defense and supporting the idea of a previously unknown protective mechanism of allergic response (35).

In humans, MCs have been involved, through different PRRs in the activation that triggers the release of pro-inflammatory mediators by different bacteria, including *Streptococcus pneumoniae*, *Staphylococcus aureus*, *Mycobacterium tuberculosis*, and *Chlamydia trachomatis* (36–39). In this case, the release of MC mediators is IgE-independent. Other immune system components, such as IgG, some cytokines, chemokines, and complement components, can directly stimulate MC degranulation (40).

A new key player in MC activation in bacterial clearance has recently been identified. Pundir et al. found that a bacterial-derived peptide could stimulate MCs through the Mas-related G protein–coupled receptor (MRGPRX2/b2). This finding demonstrates that a bacterial metabolite can directly trigger MC degranulation (36, 41). In 2015, McNeil found that mastoparan, a peptide derived from wasp venom, was capable of activating MCs by triggering their degranulation through MRGPRX2/b2 with no IgE intervention (42). Using the *S. aureus* mice infection model, Arifuzzaman demonstrated the important role of innate and adaptive immunity activated by MCs through the MRGPRX2/b2 receptor, which generates IgG immune memory (43). Detrimental effects associated with the allergen mechanism, such as anaphylaxis, represent a systemic response; however, the local effect of MCs in inflammation and clearance of bacterial infection is not IgE-mediated but is a promising therapeutic target. Other MC activation mechanisms unrelated to IgE mediated by Fc receptors have been found. In the case of IgG antibodies, they interact with the high-affinity receptor (FcγR). In humans, MCs FcγRI [inducible by interferon-gamma (IFN-γ)] and FcγRIIa are expressed, whereas, in mouse MCs, FcγRIIIa and FcγRIIb are expressed (44).

**Figure 1:**
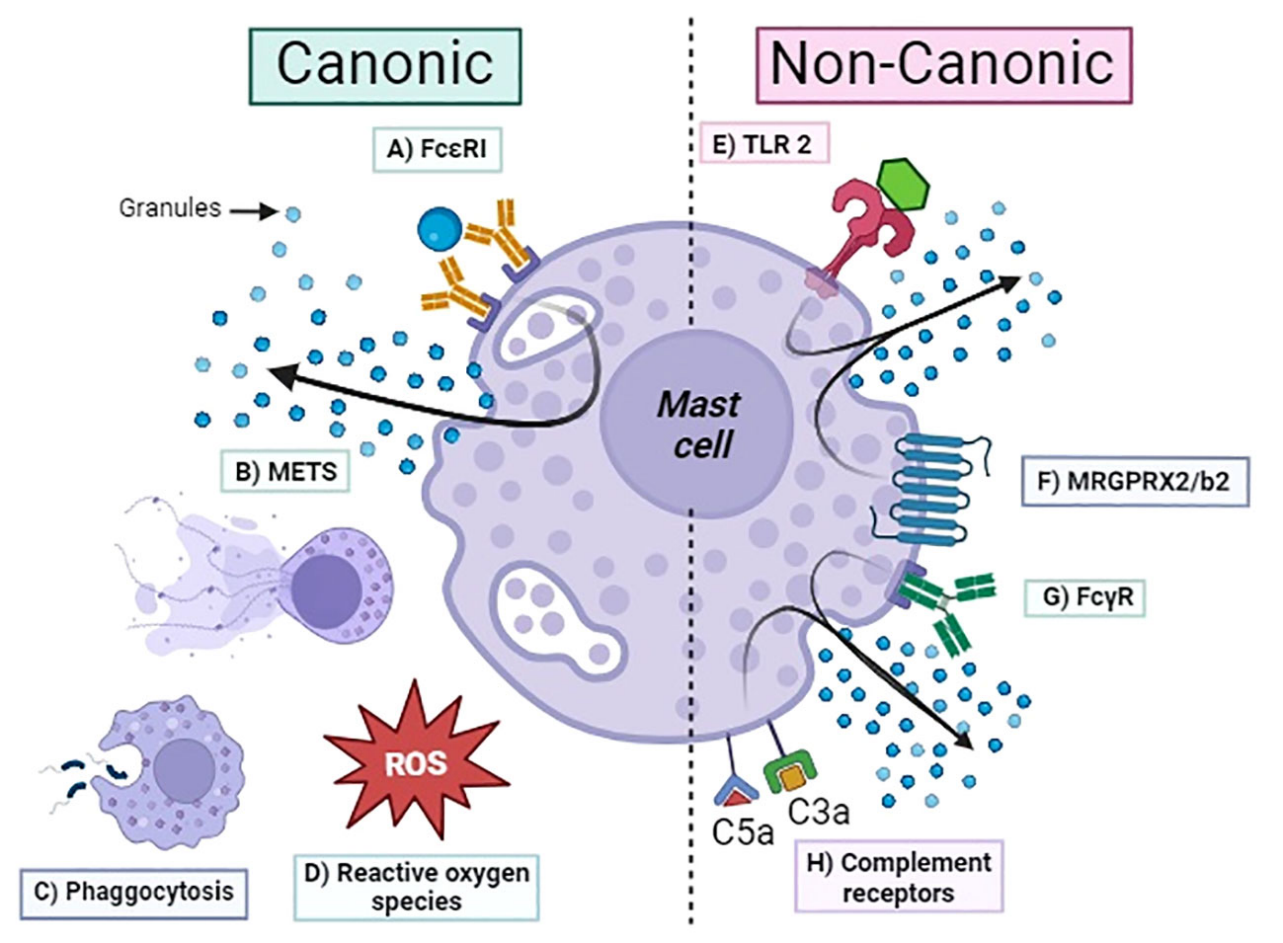
Activation mechanism of mast cells. Canonic: **(A)** FcϵRI, high-affinity IgE receptor; **(B)** METS, mast cell extracellular traps; **(C)** phagocytosis; **(D)** ROS, reactive oxygen species; **(E)** TLR2, Toll-like receptor; **(F)** MRGPRX2/b2, Mas-related G protein–coupled receptor; **(G)** FcγRs, Fc-gamma receptor for IgG; **(H)** C3aR/C5aR complement receptors.

Activation of the complement cascade occurs in the local site of tissue inflammation, where MCs typically accumulate. The demonstration that complement receptors participate in MC activation mechanisms independently of IgE highlights the relevant role of innate immunity in inflammation. C3a and C5a induce degranulation and chemotaxis in human MCs (45). Lubbers et al. in 2017 demonstrated that MCs produce C3 and, through tryptase and chymase, generate C3a, proving the prominent role of MCs in initiating local inflammation (46). MCs amplify this cross-talk by producing complement proteins and activating them through released tryptase in a feedback loop (47). These findings demonstrate the key role of MCs in homeostasis and help us understand its physiological involvement beyond the pathology association with allergic reactions. In **Figure 1**, we summarized the principal canonical and non-canonical mechanisms for MC activation.

Various sensing molecules for microbes illustrate the importance of MCs in initiating inflammation by non-allergic mechanisms. The paragraph below describes the receptors that mediate activation with or without degranulation.

## Mast cell PRR molecules sense the presence of pathogens

MCs express a range of pattern recognition receptors (PRRs) sensors that identify a wide variety of molecular patterns associated with pathogens (PAMPS) or host molecules named damage-associated molecular patterns. It is accepted that PRRs are a growing family of molecules that are located on the surface, the cytoplasm, the nucleus of cells of the innate immune response, and other non-immune cells. The demonstration of these sensing molecules in MCs located at interfaces with the environment, for example, skin, mucosa of respiratory, gastrointestinal, and urogenital, helps us understand the crucial role of these cells in the immunosurveillance process. The Toll-like receptors (TLRs) are the most studied among the family of these sensing molecules. We also include other family members such as C-type lectin-like receptors (CLRs), retinoic acid–inducible gene I (RIG-I)–like receptors (RLRs), nucleotide-binding oligomerization domain (NOD)–like receptors (NLRs), and cytosolic DNA sensor Cyclic Guanosine Monophospate-Adenosine Monophosphate (GMP-AMP) synthase (cGAS). **Table 1** presents a brief list of PRR molecules in MCs that interact with some pathogens. To better understand this complex interaction, we briefly describe the updated information to remark on the important role of MCs in initiating inflammation by known innate immunological mechanisms.

**Table 1:** Summary of innate PRRs present in mast cells and their ligands.

Receptor family	Specific receptor	Receptor ligand
Toll-like receptors (TLR)	TLR 1 (48)	Lipopeptides
	TLR2 (48, 49)	Peptidoglycan, zymosan, and some types of lipopolysaccharides
	TLR3 (50)	Double-stranded RNA
	TLR4 (49, 51)	LPS
	TLR6 (52)	Peptidoglycan and zymosan
	TLR9 (53, 54).	Bacterial DNA CpG-containing DNA
C-type lectin-like receptors (CLRs)	Mannose receptor (Group 1) (10)	Mannose, *N*-acetylglucosamine, and fucose
	Macrophage galactose-type lectin (Group 2) (10)	N-acetyl D-galactosamine
	Dectin 1 (Group 2) (55)	β-(1,3) glucans
	Dectin 2 (Group 2) (9, 56)	mannose glycoconjugates
Retinoic acid-inducible gene I like receptors (RIG-I)	RIG-I (52, 57).	Viral RNA
	Melanoma differentiation-associated factor 5 (MDA5) (52, 57)	Viral RNA
Nucleotide-binding oligomerization domain-like receptors (NOD)	NOD2 (58)	Minimal motif muramyl dipeptide (MDP)
	NOD1 (59)	Peptidoglycans
Cyclic GMP-AMP synthase (cGAS),	Cyclic GMP-AMP synthase (cGAS) (37, 60, 61).	Exogenous cytoplasmic DNA

TLRs are transmembrane receptors whose ligands interact with the different molecular pattern-associated pathogens (PAMPS). The type of ligand that activates a diversity of TLRs present in MCs has also been determined, such as TLR1− lipopeptides (48); TLR2 that recognizes peptidoglycan, zymosan, and some types of lipopolysaccharide (LPS) (48, 49); TLR3 that binds to double-stranded RNA (50); TLR4 that is activated by LPS (49); TLR6 that binds to peptidoglycan and zymosan (53); and TLR9 that recognizes bacterial DNA and CpG-containing DNA (54). MCs derived from cord blood (CBMC) consistently exhibit the TLR4 and TLR2 mRNA expression and mature protein. Interestingly, it is worth noting that, whereas TLR2 agonists stimulate the degranulation of human MCs, TLR4 agonists do not (51).

Other PRRs molecules are CLRs, which are membrane-associated and form a family of transmembrane signaling proteins with two primary groups of CLRs, each containing subgroups of receptors. Group I CLRs encompass the Mannose Receptor family, whereas Group II CLRs comprise the asialoglycoprotein receptor family. These were detected for the first time on the surface of MCs in *Bordetella pertussis* MC interactions (10). Dectin 1/CLEC7A activation elicits leukotriene release and reactive oxygen species production (55, 56), and Dectin 2/CLEC6A plays a crucial role in host defense against *Candida albicans* by promoting the differentiation of Th17 cells (52). Dectin 2 is involved in antifungal defense strategies and can function with other PRRs, such as TLRs, in various immunological processes (9). These CLRs demonstrate that pathogens such as bacteria and fungi activate MCs in mice; however, more studies are needed to elucidate if similar findings exist in humans.

Another group of pathogen-like viruses are recognized by PRR molecules, including RLRs. This group of receptors comprises a cluster of RNA helicases that serve as sensors for viral RNA in the cytoplasm. The RLR family comprises three known members: a) RIG-I, b) melanoma differentiation–associated factor 5 (MDA5), and c) laboratory of genetics and physiology 2 receptors (52). Studies using small interfering RNA analysis to identify the receptors responsible for MC activation by vesicular stomatitis virus demonstrated that both RIG-I and MDA5 are involved in cytokine production but do not play a role in the degranulation of MCs, contributing to antiviral responses (57).

The fourth group of PRR molecules located in the cytoplasm of MCs and senses gram-positive and negative bacteria are the cytosolic NLRs. It was published that this group includes four subfamilies: a) acidic transactivation domain, b) baculoviral inhibitory repeat-like domain, c) CARD domain, and d) pyrin domain (9). PAMPs like the peptidoglycans from gram-negative and gram-positive bacteria can be recognized by NOD2 through the detection of the minimal motif muramyl dipeptide (MDP) activating nuclear factor-kB (NF-kB) in the human MC line (HMC-1) (58). *In vivo* experiments have demonstrated that MMCs play a pivotal role in peptidoglycan (PGN)–induced diarrhea. It has been established that TLR2 facilitates the absorption of PGN into MCs. Once inside the cytoplasm, PGN activates NOD1, subsequently triggering MC activation. A synergistic effect between histamine and serotonin released by MCs appears to contribute to PGN-induced diarrhea (59). As we can see, most of the information about PRR’s role in MC activation has been obtained in mice and cannot necessarily be extrapolated to humans. However, this limitation is because MCs were only studied in an allergic context. Additional studies are urgently needed to understand if these mechanisms are affected or influenced when an IgE antibody concentration is present.

The final group included in this review is a cytosolic DNA sensor known as cGAS. This member has recently been described in the context of cancer immunity, pathogen infections, and autoimmune diseases by initiating the production of IFN type I (IFN-I) (60). Normally, cGAS remains inactive within the cell and becomes active when it binds to irregular DNA. Once activated, cGAS generates 2′,3′-cGAMP, which functions as a secondary messenger, triggering the activation of a stimulator of IFN genes. This secondary messenger begins a signaling cascade activating tank-binding kinase 1 that activates type I IFN regulatory factor 3, culminating in the expression of type I IFNs and inflammatory cytokines in collaboration with NF-κB (61). In the human-derived MC line known as HMC-1, it was demonstrated that *S. aureus in vitro* infection activates the cGAS signaling pathway described above, leading to the generation of IFN-I. It is crucial to emphasize that the live bacterium is necessary for initiating IFN-I production because using heat-killed bacteria completely halts IFN-I production by HMC-1 cells (37).

The nature of the molecule that stimulates the ligand-receptor determines the signaling pathway that may lead to the release of various response factors, including cytokines, chemokines, and pro-inflammatory mediators, each of which can have diverse physiological effects, ranging from functions including immunomodulation and antimicrobial activity.

According to the cited literature, most of the information obtained in mice helped us understand the biological importance of MCs as a major innate immunity component. However, we know this information needs to be demonstrated in healthy people and allergic pathology contexts. Potential new therapeutic targets can be of help as immunomodulatory benefits of activating or inhibiting MCs in the context of allergy, infection, and other diseases.

## Immunomodulation by mast cells

MCs are especially effective in immune vigilance because they have certain inherent properties, such as their strategic location at the host–environment interface, including the skin and mucosa, allowing them to be the first line of pathogen recognition when the integrity of these barriers is compromised (16). However, in an infectious context, their functions go beyond hand-to-hand combat because MCs can trigger an immune response that involves other cell populations to contribute to the control and subsequent elimination of the pathogen. MCs are filled with a wide spectrum of receptors and costimulatory molecules that have immunoregulatory functions and represent a potential source of many potent chemical mediators, such as growth factors, chemokines, and cytokines, some of which can be rapidly released upon MC activation (17). There are various mechanisms by which MCs can modulate the immune response, ranging from enhancing or suppressing the development, survival, recruitment, activation, maturation, and proliferation of innate and adaptive immune cells. Different mechanisms of MCs and other immune cells cross-talk; for example, a study on chemotactic cytokine production showed that incubation with monomeric IgE increases the production of chemokines by human MCs, such as monocyte chemoattractant protein-1 (MCP-1). This effect was more pronounced when cells were preincubated with interleukin-4 (IL-4). Although MCP-1 may not be a strong chemoattractant for MCs, it has been demonstrated to be a potentially significant mediator of eosinophil, monocyte, and CD4+/CD8+ lymphocyte recruitment (62). Another interaction documented between locally activated MCs and peripheral CD4+ T cells is the recruitment of T helper cells by C-C motif chemokine ligand 1 (CCL1) (63), CCL3, and CCL 4 (64). It is crucial to emphasize that the goal of cell recruitment is to aid in the clearance of pathogens, and the nature of the stimulus can determine whether the infection is successfully contained. This event is evident when comparing three infection models with *Listeria monocytogenes*, *Escherichia coli*, or *Staphylococcus aureus*. In these infections, MCs release mediators independent of degranulation that are bacterial specific. These authors found higher levels of IL‐8, MCP‐1, and prostaglandin D2 (PGD2) released by *L. monocytogenes* stimulus than *E. coli*. In the case of *S. aureus*, this bacteria does not trigger the release of the mentioned mediators and induces PGD2 secretion at significantly lower levels (65).

The nature of the stimulus determines the type of MC response and the subsequent immune modulation effect; however, for chemotactic molecules released to exert their effects, there are necessary changes in the local microenvironment. Therefore, once a specific response is initiated, a cascade of multiple physiological functions is set to promote cell recruitment at the local level. Circulating cells must traverse blood capillaries to reach the inflammation or infection site without causing damage. This process is facilitated by an increase in vascular permeability mediated by vascular endothelial growth factor via modulation of phosphatidyl inositol 3 kinase–hypoxia-inducible factor-1 (66) and histamine (67) released by MCs. The strategic localization of MCs near blood vessels ensures a rapid impact on both blood and lymphatic vessels shortly after MC activation (68). During this journey, MCs also promote an increase in the expression of adhesion molecules in the vascular endothelium, such as intercellular adhesion molecule-1, vascular cell adhesion molecule 1, P-selectin, and E-selectin, through the action of cytokines such as tumor necrosis factor–alpha (TNF-α), IFN-γ, and IL-6 (69–71). Simultaneously, leukocytes undergo an activation process influenced by various cytokines and pro-inflammatory mediators released by MCs, including TNF-α (72, 73), IFN-γ, and prostaglandin E2 (74, 75). The primary aim of adhesion and activation is the trigger of various cell types like granulocytes, monocytes/macrophages, dendritic cells (DCs), natural killer (NK), lymphocyte T helper (Th), and lymphocyte B, among others, to quickly initiate a widespread local and systemic response.

Upon stimulation of MCs, they initiate neutrophil recruitment by releasing the chemokine C-X-C motif ligand CXCL1/CXCL2 (76). Simultaneously, the physiological events associated with neutrophil recruitment begin following the release of MC pro-inflammatory mediators. This sequence of events includes a) intravascular rolling along the luminal blood vessel endothelium, b) firm adhesion, c) crawling, and d) diapedesis through the vessel wall (77). Throughout this process, neutrophils are stimulated by MC-derived TNF, which is released early, along with the mentioned chemokines. This cytokine is crucial in facilitating the extravasation and activation of neutrophils, as demonstrated in experiments involving mice lacking the TNF receptor R1 (*Tnfrsf1a−/−*) (73). These mice exhibited a phenotype characterized by a reduction in the number of neutrophils infiltrating the skin.

Additionally, there was an increase in blood neutrophils, primarily with lower CD11b expression, and a failure to upregulate the surface expression of macrophage-1 antigen (Mac-1) as well as Ly6G in mouse MCΔTnf (73, 78). Indeed, the influence of MCs on neutrophils extends beyond their recruitment. Evidence demonstrates that MC tryptase is associated with forming neutrophil extracellular traps (NETs) through a mechanism resembling neutrophil elastase activity. This finding involves the ability of tryptase to cleave off the N-terminal ends of core histones, contributing to DNA decondensation and promoting NET formation (79).

Other modulated cells as monocytes and macrophages by MCs were demonstrated in a study that showed that peritoneal macrophages from Kit(W^-sh^/W^-sh^) and WT mice were exposed to dengue virus (DENV). They found that there was a tendency for higher DENV infection and increased secretion of CCL2 (MCP-1) in peritoneal macrophages from Kit(W-^sh^/W-^sh^) mice compared to those from WT. In the same publication using *in vivo* experiments, these authors demonstrated that intradermal DENV inoculation in Kit(W-^sh^/W-^sh^) mice showed elevated levels of infiltrating macrophages and CCL2 (MCP-1) at the inoculation site (80). The atypical increase in MCP-1 and macrophages observed in the infection model described for Kit(W-^sh^/W-^sh^) mice can be attributed to the absence of proteases. This phenomenon was elucidated in another study. It was demonstrated that tryptase released from human cord blood–derived MCs following FcϵRI cross-linking could degrade MCP-1 co-released during MC activation (81). In another publication using *Cryptococcus neoformans* (82), authors found similar results, confirming that the interaction with monocytes depends on the nature of the offending microbe; in this case, the sensing of *Cryptococcus neoformans* by MCs triggers the release of inflammatory mediators, including tryptase and chemokines such as CCL-2/MCP-1. Notably, CCL-2/MCP-1 functions as a robust and selective chemoattractant for monocytic cells. In this scenario, supernatants from infected MCs attracted monocytes and not neutrophils.

Histamine is one of the most important MC mediators with various immunomodulatory properties. One of these prompts the differentiation of dermal DCs into a subset CD1a−CD14+, even in the presence of IL-4 and granulocyte-macrophage colony-stimulating factor (GM-CSF). This population, CD1a−CD14+, exhibits a decreased capacity to stimulate resting T cells due to a diminished ability for antigen presentation, in contrast to the other two subsets: CD1a−CD14− and CD1a+CD14−. Additionally, this DC subpopulation with the CD1a−CD14+ phenotype displays an increased capability to stimulate the production of pro-inflammatory cytokines and chemokines, coupled with heightened phagocytic activity (83). Another unique reported property of MCs is that they affect lymphoid tissue–borne adaptive immunity over distance by modifying DC functionality by delivering granule-stored mediators. DCs engulf the intact granules exocytosed by MCs during skin inflammation, and the engulfed MC granules are actively shuttled to skin-draining lymph nodes and finally degrade inside DCs within the lymphoid tissue. Most importantly, phagocytosed granules derived from MCs promote DC maturation and migration to the skin-draining lymph nodes, partially through MC-derived TNF, and boost their T-cell priming efficiency (84).

MCs modulate adaptive immunity by modifying T-cell responses through at least one known mechanism. The direct cross-talk between these cells involves OX40L on the MCs surface, inducing T-cell proliferation and their survival in an OX40L-dependent manner (64). This interaction enhances T-cell proliferative responses to various mitogens such as phorbol myristate acetate, phytohemagglutinin, and anti-CD3 (85). Modulation of T helper cells by MCs occurs by the same molecular mechanism through OX40 and OX40L. This interaction has been shown to induce MC degranulation by increasing cyclic adenosine monophosphate and reducing Ca^2+^ influx. This response is independent of phospholipase Cγ or Ca^2+^ release from intracellular stores, reducing the immediate hypersensitivity response (86, 87). In addition, other molecules that mediate MC modulation functions have been described after stimulation with TNF-α that upregulates an inducible costimulator ligand (ICOSL) expression. Co-culture of ICOSL+ MCs and CD4+ T cells revealed changes in the percentage of regulatory T cells. Briefly, TNF-α–treated MCs promoted CD4+ T-cell differentiation into CD4+CD25+Foxp3+ T cells via ICOSL/ICOS interaction. Furthermore, they observed changes in the secretion of cytokines linked to Th1 and Th2 immune responses in supernatants collected from the co-culture system. Notably, there was a decrease in the levels of the Th1 marker cytokine IFN-γ, accompanied by a simultaneous increase in the expression of Th2 marker cytokines, namely, IL-4 and IL-10 (87).

Tissue CD8 T cells interact with MCs, resident memory T cells, and T CD8 recirculating cells. One example of this cross-talk occurs through the activation of an IgE/FcϵRI interaction, which can selectively induce the migration of CD8 effector memory T cells mediated by leukotriene B4 (88). Other immunomodulatory effects of MCs are the activation and proliferation of antigen-specific CD8+ T cells through direct cell-to-cell contact and major histocompatibility complex class I–dependent antigen cross-presentation by MCs. Consequently, CD8+ T cells respond by secreting IL-2, IFN-γ, and macrophage inflammatory protein–1α (89).

**Figure 2:**
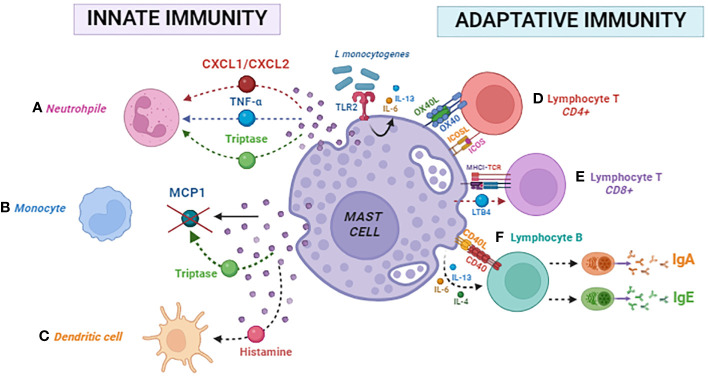
Mast cell modulates innate and adaptative immunity. Innate immunity: **(A)** neutrophil, **(B)** monocyte, and **(C)** dendritic cell. Adaptative immunity: **(D)** CD4 lymphocyte, **(E)** CD8 lymphocyte, and **(F)** B lymphocyte.

Different physiological functions have demonstrated MCs’ immunomodulatory effects on B cells. In the presence of cytokines secreted by activated MCs, such as IL-4, IL-13, or IL-6, B cells undergo activation through the CD40–CD40L interaction via the TGase 2 enzyme (90). This activation, facilitated by pathways involving TRAF2–MEKK1 and TRAF6–TAK1, promotes Ig class-switching. Consequently, B cells are directed toward producing immunoglobulin IgE and IgA. In addition, OX40–OX40L interaction, combined with cytokine stimulation, activates B cells through a signaling pathway involving TRAF6–TAK1, leading to the production of IgE and IgA. Notably, IgE and IgA produced by these B cells can initiate a feedback loop by re-activating MCs. This process occurs by binding IgE to FcϵRI and IgA to FcαRI on MC surfaces. This reactivation triggers further mediator release, contributing to the amplification and maintenance of the allergic immune response in mice (91). In **Figure 2**, we summarized the innate and adaptive immunomodulatory mechanisms of MCs mentioned above.

## Role of mast cells in bacterial infections

MCs exert a protective role in controlling bacterial infections, as shown by *in vitro* and *in vivo* studies through indirect mechanisms (34, 43, 92–95). One is IL-6 release by MCs that boost the host’s innate immune response, which induces antimicrobial peptide production and enhances the antibacterial capacity against *Pseudomonas aeruginosa* in mouse keratinocytes. MCs produce a variety of cytokines in response to a bacterial stimulus; the most commonly observed activation mechanism, independent of the classic IgE-mediated activation, is centered on PRRs. An example of this is mediated by TLR2 and several bacterial stimuli, such as *Listeria monocytogenes* infection, where this receptor is crucial for producing IL-6 and IL-13 (34). In the case of *Mycobacterium tuberculosis*, the 19-kDa Mtb lipoprotein promotes the release of IL-8, MCP-1, and GM-CS (38).

In the case of Chlamydial infections, it has been published that *Chlamydia trachomatis* promotes the release of IL-1RA, IL-23, CCL3, GM-CSF, CCL5, and CXCL8. Similar information was obtained with *Chlamydia muridarum* stimulus, which induces the secretion of CXCL2 and IL6 in BMMCs infected under the interaction of the major outer membrane protein (MOMP) binding with TLR2 (39). Recently, other authors found similar results with the *Brucella abortus* bacterium. In this publication, they show that the activation of TLR2 and TLR4 by outer membrane protein and LPS, respectively, induces a cell signaling cascade and the production of IL-6, TNF-α, CCL3, CCL4, and CCL5 (96).

Extracellular bacteria such as *Staphylococcus aureus* were shown to promote MCs activation and secretion of cytokines by other TLR-independent mechanisms; in this publication, authors demonstrated that the production of IFN-I involves the activation of cGAS (61). *Streptococcus pneumoniae* in a murine nasopharyngeal infection model with other extracellular bacterial promote MC activation through the stimulation of Mas-related G protein–coupled receptors (GPCRs) in mice (Mrgprb2) by the competence-stimulating peptide–1 (CSP-1) (bacterial quorum–sensing molecule), leading to the recruitment of neutrophils and secretion of TNF, PGD2, and ROS (36).

*Streptococcus equi*, another extracellular bacterium, can also directly activate MCs that help recruit monocytes, DCs, and effector T cells to infection sites, contributing to bacterial clearance. The co-culture of *S. equi* with BMMCs promoted a profound secretion of CCL2/MCP-1, CCL7/MCP-3, and CCL5/RANTES (97).

These examples of intracellular and extracellular bacteria demonstrate that the nature of bacterial infection influences the MCs’ direct activation and highlights the important role of these innate immune cells in initiating the local inflammation that later, through different mechanisms, recruits other cells, enhancing inflammation that contributes to bacterial clearance. **Table 2** summarizes the updated known MC mechanisms that contribute to the activation of innate and acquired immune responses.

**Table 2:** Mast cell receptors involved in antibacterial mechanism.

MC receptor	Mast cell source	MCs receptor ligands	Bacterial stimuli	MCs mediators	Host	Reference
TLR2	PBMCs	19-kDa lipoprotein	*Mycobacterium tuberculosis*	IL-8, MCP-1, GM-CSF, and IL-13	Human	(38)
	CBMCs	MOMP	*Chlamydia trachomatis*	TNF, IL-1RA, IL-6, IL-23, CCL3, GM-CSF, CCL5, and CXCL8	Human	(39)
	BMMCs	MOMP	*Chlamydia muridarum*	CXCL2 and IL6	Mice	
	BMMCs	OMP	*Brucella abortus*	IL6	Mice	(96)
TLR4	BMMCs	LPS	*Brucella abortus*	TNF-α, CCL3, CCL4, and CCL5	Mice	
cGAS	HMC-1	c-di-AMP	*Staphylococcus aureus*	IFN-I (IFN-α)	Human	(61)
Mrgprb2 MRGPRX2	CTMCs LAD2	CSP-1	*Streptococcus pneumoniae*	TNF, ROS, and PGD_2_	Mice; human	(36)
CD48	BMMCs	FimH-Type 1	*Escherichia coli*	TNF-α	Mice	(95)
MR MGL	BMMCs PCMC	LOS	*Bordetella pertussis*,	TNF-α, IL-6, and IFN-γ	Mice	(10)

MCs’ complexity with many PRRs on their surface and the plasticity of their response mediated by different mechanisms, including the release of preformed mediators and new produced like cytokines, chemokines, and other molecules, highlights their pivotal role in homeostasis.

## Mast cell as a new therapeutic target

In the previous sections, it is clear that MCs’ role in homeostasis is mediated by at least three different mechanisms: a) IgE canonical activation; b) PRR noncanonical, IgE independent; and c) MRGPRX2/b2 cationic peptide activation.

MAS-related G protein–coupled receptor-X2 (MRGPRX2) in humans and its ortholog MAS-related G protein–coupled receptor-b2 (Mrgprb2) in mice belong to the δ-branch of the rhodopsin-like class A of GPCRs and constitute the largest non-odorant family of seven-transmembrane receptors (98). MRGPRX2/b2 can primarily be activated by cationic substances, including neuropeptides, bacteria-derived quorum-sensing molecules (QSMs), venom peptides, host defense peptides (HDPs), and various FDA-approved drugs (99). It is well-documented that MRGPRX2/b2 can activate MC, causing pseudo-allergic drug reactions, neurogenic inflammation, pain, itch, and chronic inflammatory diseases such as urticaria and asthma (100).

In recent years, the MRGPRX2/b2 receptor has attracted significant interest as a potential therapeutic target owing to its pseudo-allergic mechanism. Identifying novel molecules capable of selectively activating or modulating MCs through MRGPRX2/b2 can help clear infections as an alternative to antibiotic therapy. It is important to emphasize the unique feature of MRGPRX2/b2 that distinguishes it from other GPCRs, its selective expression. Specifically, human MRGPRX2 and mouse Mrgprb2 are found exclusively in tryptase and chymase MCs (MCTC) and connective tissue MCs (CTMC) (36, 42, 101).

The skin is an anatomical site prone to bacterial infections; however, the maintenance of immunosurveillance is largely attributed to MCs. Some studies have explored novel strategies by stimulating MRGPRX2/b2 in this organ to enhance the protective effects of MCs in skin infection models.

Mastoparan is a cationic α-helical peptide derived from *Vespula lewisii* venom that activates MCs through Mrgprb2 and has been used topically in *Staphylococcus aureus* from the infected mouse skin. This activation enhances the clearance of the infection and expedites the healing of dermonecrotic lesions. This effect is achieved by recruiting bacteria-clearing neutrophils and wound-healing CD301b+ DCs. Additionally, there is increased migration of antigen-presenting DCs to draining lymph nodes, providing stronger protection against a subsequent infection challenge (43). Other molecules that can trigger degranulation in human MCs by activating Mas-related proteins are HDPs (101). Examples include LL-37 (102), human β-defensin 2, and human β-defensin 3, which activate MCs and induce degranulation via MRGPRX2 (103). However, HDPs are metabolically unstable and display cytotoxicity, which limits their clinical utility. A series of small-molecule HDP mimetics have been developed to address these constraints (104). These compounds can be synthesized in a significantly shortened, stable, and less toxic form while preserving or enhancing their antimicrobial properties. Several synthetic mimics are currently under clinical development for various infectious diseases (105). Murepavadin, or POL7080, is a lipidated HDP mimetic with antibacterial activity against a broad spectrum of multidrug-resistant *P. aeruginosa*, contributing to bacterial clearance and promoting wound healing. It induces Ca^2+^ mobilization, degranulation, and production of chemokines IL-8 and CCL3 in a human MC line (LAD2 cells) that endogenously express MRGPRX2. In mouse peritoneal MCs, Mrgprb2 induces vascular permeability in WT mice but not in Mrgprb2−/− mice (106). In this study, compound 48/80 was tested to observe induced β-arrestin recruitment (participation in agonist-mediated desensitization of GPCRs) (36, 107) and promoted receptor internalization, which resulted in a substantial decrease in the subsequent responsiveness to the MRGPRX2 agonist, causing a specific shutdown of the cellular response.

Another strategy involving the activation of MRGPRX2/b2 is using QSMs as an “Achilles heel.” One of these molecules is CSP-1 of *Streptococcus pneumoniae*, which acts as a ligand for both Mrgprb2 and MRGPRX2 receptors. For MRGPRX2 in a human MC line, such as the Laboratory of Allergic Diseases 2 (LAD2), there has been an increase in ROS, TNF, and PGD2, inhibiting bacterial growth and preventing biofilm formation. Mrgprb2 enhances the production of TNF, along with an increase in neutrophils in nasal lavage fluid, resulting in a subsequent decrease in bacterial growth (36).

The recent results mentioned above promise to offer valuable insights into the involvement of MCs in addressing bacterial infectious disease control. The activation of MCs through MRGPRX2/b2, due to its crucial role in combating infections, represents a great opportunity to investigate its direct and indirect elimination of bacterial pathogens.

Agonist molecules that activate MCs through the MRGPRX2/b2 may have future use in therapy as immunomodulatory of innate and adaptive immunity host response to control infections.

## Discussion and future perspectives

The abundance of evidence documenting, supporting, and substantiating the involvement of MCs in the pathological mechanisms of allergies is overwhelming, leaving no room for doubt about their central role. However, in recent years, we have seen the emergence of new evidence regarding the existence of non-canonical receptors and mechanisms of MCs activation, degranulation, as well as non-canonical processes, suggesting that the mechanism of IgE–FcϵR is not the central mechanism of mediator release. The nature of the infection determines the activation type that eliminates the pathogen. These non-canonical mechanisms put into perspective the antimicrobial potential of MCs beyond their role in allergic pathology.

The high quantity of stored and the *novo* synthesis of pro-inflammatory mediators include histamine, cytokines, chemokines, neurotransmitters, growth factors, antimicrobial peptides, lipid mediators, and other substances, all modulate the immune response during infection.

This immunomodulating mechanism by MCs goes beyond soluble mediators and also includes cell to cell interaction and communication with innate and adaptative immune cells. The signaling effect influences not just at the local level but also the difference of canonical degranulation with the anaphylactic detrimental known effects. Some microorganisms triggered some non-canonical degranulation mechanisms. MCs’ antimicrobial effect represents an opportunity to develop new therapeutic targets to modulate immune host response enhancers and antibiotic therapy to control human infections.

Limiting the excessive acute inflammation in some infections may help the host to limit tissue damage; thus, targeting MCs as a source of pro-inflammatory cytokines can be studied in detail in different human pathologies.

As mentioned above, we want to remark that MRGPRX2/b2 represents a good example of a new promising therapeutic target for selective activation or modulation of MCs. In recent publications, it was shown that agonist cationic peptides trigger the degranulation mechanism of these cells without a detrimental effect.

Despite considerable efforts to explore the nature and function of MCs beyond their involvement in allergic pathology, many questions remain unanswered. New investigations are needed to dissect the mechanisms, the molecules, genes, and their complex interactions that ultimately lead to bacterial clearance, contributing to our understanding of the important biological role of these MASTer cells.

## Author contributions

MCSC: Writing – review & editing, Conceptualization, Data curation, Supervision, Formal analysis, Validation, Investigation. TASV: Writing – review & editing, Writing – original draft, Validation, Supervision, Formal analysis, Investigation, Data curation, Conceptualization, Methodology. NL-L: Writing – review & editing, Writing – original draft, Supervision, Project administration, Investigation, Conceptualization.
